# Metaphor analysis meets lexical strings: finetuning the metaphor identification procedure for quantitative semantic analyses

**DOI:** 10.3389/fpsyg.2023.1214699

**Published:** 2023-11-08

**Authors:** Laurence De Backer, Renata Enghels, Patrick Goethals

**Affiliations:** ^1^Department of Linguistics, Ghent University, Ghent, Belgium; ^2^Department of Translation, Interpreting and Communication, Ghent University, Ghent, Belgium

**Keywords:** metaphor analysis, metaphor identification, lexical units, lexical strings, unit boundary demarcation, quantitative analysis, semantic field analysis, methodology

## Abstract

Recent years have witnessed the development of the Metaphor Identification Procedure (MIP/VU), a step-by-step protocol designed to identify metaphorically-used words in discourse. However, MIP(VU)‘s merits notwithstanding, the procedure poses a problem to scholars intending to use its output as the basis for a semantic field analysis involving a quantitative component. Depending on the research question, metaphor analysts may be interested in chunks of language situated above the procedure’s standardized level of analysis (i.e., the lexical unit or lexeme), including phrases and sentences. Yet, attempts to decenter the method’s exclusive focus on metaphor-related words have been the target of critique, among others on the grounds of their lack of clear unit-formation guidelines and, hence, their inconsistent unit of analysis and measurement. Drawing on data derived from a Spanish-language US-based newspaper’s coverage of the migration program known as DACA (Deferred Action for Childhood Arrivals), this article describes challenges that analysts can run into when attempting to use a dataset containing atomized metaphor-related words as the input for subsequent quantitative semantic analyses. Its main methodological contribution consists in a proposal and illustration of three possible methods to extend the existing MIP(VU)-protocol in such a way as to allow it to capture metaphorical strings, on top of lexemes, in a reliable and systematic manner. The first two methods are procedural, and entail formulating *a-priori* grouping-directives based on the research question(s). One departs from semasiological criteria (Method 1) and the other takes an onomasiological approach (Method 2). The third method works bottom-up, involving the *ad hoc* grouping of lexemes and adding a descriptive parameter meant to keep track of grouping-decisions made by the analyst, thereby safeguarding transparency at all times.

## 1. Introduction

The cognitive turn of the 1980s ushered in an era of intensified (Psycho)Linguistic interest in metaphors (e.g., Ortony, 1993; Kövecses, 2010). As the popularity of metaphor research surged, a concern with developing an empirical method for detecting metaphors in naturally occurring speech and writing ensued. This concern was fed by a number of methodological critiques leveled to existing Conceptual Metaphor scholarship, which had tended to use pre-established lists of conceptual metaphors to identify linguistic expressions taken to instantiate them, thereby becoming vulnerable to confirmation bias (Krennmayr, 2013). In the wake of these reproaches, various initiatives to systematize metaphor identification stepped up to the challenge,^1^ among which one of the most impactful one has been the Metaphor Identification Procedure (Pragglejaz Group, 2007).

The Metaphor Identification Procedure – or MIP for short – entails a step-by-step protocol designed to identify metaphorically-used words in discourse. It was created by Pragglejaz Group (2007) and further refined under the acronym “MIPVU” (Steen et al., 2010). In brief, the procedure requires the analyst to establish for individual lexical units whether they possess a more basic, concrete sense than their contextual one, and whether both meanings can be understood in contrast and comparison with one another (see Section 2.1). If the response is affirmative, the unit is marked as metaphorical. Contrary to top-down approaches to metaphor analysis, MIP(VU) works from the language data upwards (Krennmayr, 2013) and reserves the semantic annotation of metaphor mappings (e.g., SOCIETY IS A BODY) for a consecutive stage of analysis (Pragglejaz Group, 2007; Steen et al., 2010, p. 8; Krennmayr, 2013; Steen, 2017).

However, MIP(VU)‘s^2^ merits notwithstanding, the procedure also poses different challenges. The focus of this paper is concerned with the output of MIP(VU) as the basis for quantitative semantic analyses, which are far from evident (also see Glynn, 2010; Jansegers et al., 2015). Specifically, the problems for such analytic ends boil down to MIP(VU)‘s standardized unit of analysis, called the lexical unit, as relevant linguistic items do not always neatly overlap with lexemes (*cf.*, Cameron and Maslen, 2010; Charteris-Black, 2014; Perrez and Reuchamps, 2014; Silvestre-López and Navarro i Ferrando, 2017). Depending on the research question, metaphor analysts may be interested in chunks of language situated above the level of the lexical unit. These may range from compositional phrases (1) over non-compositional phrases (2)^3^ or even complete sentences (3).

(1) His mother’s death hit him hard^4^ (Lakoff and Johnson, 1980; cited in Deignan, 1999, p. 22).

(2) [feeling] on top of the world^5^ (Pinker, 2015, p. 396).

(3) Two people are chasing the same world title^6^ (Sullivan, 2007, p. 108)

In the context of their Discourse Dynamics Framework to metaphor analysis, Cameron and Maslen (2010) developed an alternative identification procedure which is attuned to the identification of larger stretches of metaphorically-motivated language, called *vehicle terms*. An example of a multiword expression identified as a vehicle term is the phrase *flaw in the system*, the basic meaning of which can be paraphrased as “a mechanical breakdown caused by some physically incorrect item” and its contextual meaning in a text on terrorism being “failures in security service tactics and procedures” (Cameron and Maslen, 2010, p. 107).

Steen (2017), however, has voiced a methodological concern about the protocol, in particular, how to decide where vehicle-boundaries fall. First, the decision of where to place unit boundaries seems to rely on the idea of ‘metaphorical coherence’, i.e., coherence of the metaphorical image evoked by the items within the vehicle (Steen, 2017, p. 83). Problematically, this tacitly requires the analyst to postulate underlying mappings of the string’s constituent elements, thereby lumping together metaphor identification and semantic analysis, which is exactly the methodological problem that MIP(VU) was meant to avoid. Second, the method’s lack of clearly defined guidelines to orient grouping decisions translates into an inconsistent unit of analysis. And, it is well known that the arbitrary nature of unit formation brings into jeopardy the comparability of obtained quantitative results (*cf.*, Steen et al., 2010).

Against this backdrop, the aim of this paper is threefold. First, drawing on concrete examples extracted from a corpus-based discourse study on a Spanish-language US-based newspaper’s metaphorical framing of the Deferred Action for Childhood Arrivals (DACA) debate, we seek to paint a nuanced picture of the challenges that the lexical unit (which we will later refer to as ‘lexeme,’ for disambiguation’s sake; *cf.*, Section 2) can pose to metaphor analysis. Namely, what if the relevant metaphorical image for a particular research interest is triggered by a lexical string, broken up into multiple units according to MIP(VU)? The second objective consists in illustrating two methodological difficulties that metaphor scholars may encounter when using the output generated by MIP(VU) as the starting point of their quantitative semantic analysis. These methodological challenges relate to (i) the semantic annotation (e.g., WAR, DISEASE) and (ii) quantification of metaphorically-motivated strings. Specifically, how do we annotate and count their component parts? Finally, we wish to contribute to the methodological debate sketched above by proposing some guidelines to further finetune MIP(VU) for academics who (also) want to capture metaphorical expressions which stretch beyond the boundaries of the lexical unit. Concretely, we will present three alternative methods to achieve this: Method 1 and 2 are procedural, and consist in formulating *a-priori* grouping-directives based on the research question(s); while Method 1 departs from semasiological (i.e., ‘form-first’) criteria, Method 2 takes an onomasiological (‘concept-first’) approach. Method 3 is bottom-up, involving the *ad hoc* grouping of units and adding a descriptive parameter meant to keep track of grouping-decisions made by the analyst, thereby safeguarding transparency at all times.

The structure of this paper breaks down in five main parts. Section 2 will provide an overview of the different steps that make up MIP(VU), paying special attention to the lexical unit and its proposed way to deal with (non-)compositional multiword expressions and other linguistic multiword realizations of cross-domain mappings (such as similes and analogies). We do so to familiarize uninitiated readers with some of the procedure’s features and terminology, thereby laying the foundation for the remainder of the paper. Section 3 starts with a brief introduction of the case study from which the data used to illustrate our main methodological points stems (3.1), before setting out the core problem and related methodological challenges that metaphor scholars may face when holding onto atomized lexical units as the input for subsequent semantic field- and quantitative analyses (3.2). We then present three methods which allow to sidestep these difficulties in section 4, after which we offer some concluding remarks in the final section.

## 2. Introducing the MIP(VU) method

### 2.1. A brief introduction of MIP(VU)

At its core, the Metaphor Identification Procedure (MIP) comprises four main steps^7^. The first consists in reading the entire text to get a sense of its meaning as a whole. The second step requires the analyst to divide the text’s strings of verbiage into their constituent lexical units. Lexical units are generally understood as individual words (Pragglejaz group, 2008, p. 2), with the exception of multi-word clusters whose “meaning of [the] whole expression cannot be arrived at *via* the composition of the meaning of the parts” (Pragglejaz Group, 2007, p. 4). In this way, the sentence “For years, Sonia Gandhi has struggled to convince Indians that she is fit to wear the mantle of the political dynasty into which she married, let alone to become premier” would be composed as (Pragglejaz Group, 2007, p. 4):

/For / years /, Sonia Gandhi / has / struggled / to / convince / Indians / that / she / is / fit.

/ to /wear / the / mantle / of / the / political / dynasty / into / which / she / married /, let.

alone / to / become / premier / (Pragglejaz Group, 2007, p.4).

The next step involves inspecting each of these lexical units to distinguish their contextual meaning, bearing in mind the surrounding context, and to check whether there exists a more basic, concrete and/or bodily action-related interpretation. To decide on a lexical unit’s basic meaning, MIP prescribes the use of a dictionary (Pragglejaz Group, 2007).

In the last step, a lexical item is marked as metaphorical when (i) the consulted dictionary mentions a more concrete sense than the contextual one observed in the corpus and when (ii) both senses can be understood in contrast and comparison with one another. Consider the analysis that Pragglejaz Group proposes for the lexical unit *wear* taken from the example sentence (Pragglejaz Group, 2007, pp. 7–8):

wear.

“(a) *contextual meaning*: In this context, the idiomatic expression “wear the mantle” means to have a leading role within a family whose members have recently occupied positions of high office in a particular democratic system. The contextual meaning of “wear” is have or bear, and the contextual meaning of “mantle” is the familial responsibility.

(b) *basic meaning*: The basic meaning of “wear” in “wear the mantle” is defined as the first sense of the word in the Macmillan dictionary as follows: “to have something on your body as clothing, decoration or protection” (p. 1,622). The SOEDHP indicates that this meaning is also historically prior (p. 1,274).

(c) *contextual meaning* versus *basic meaning*: The contextual meaning contrasts with the basic meaning and can be understood by comparison with it: We can understand the process of following family members in having a prominent political role in terms of physically wearing the item of clothing that symbolizes royal power.

*Metaphorically used?* Yes.”

In a next phase, the MIP-identification instrument was further adapted and expanded under the acronym MIPVU (Steen et al., 2010). Probably its most pivotal extension relevant for this paper involves MIPVU’s incorporation of the analytical category of *direct metaphor*. This type is defined as language use potentially realizing a cross-domain mapping (Steen et al., 2010, p. 10), and encompasses most (metaphorical) similes (e.g., *her harlequin dress like some angry restless dragonfly*; Steen et al., 2010, p.93), analogies (e.g., *The DNA can be regarded as a set of instructions for how to make a body, written in the A, T, C, G alphabet of the nucleotides;* Dawkins, 2016, p. 28) and other explicit invitations for comparison (e.g., *Shall I compare thee to a summer’s day*; Steen et al., 2010, p. 10). This category of language use is set apart from the linguistic realizations of metaphor that MIP was designed to capture, which MIPVU’s architects have baptized *indirect metaphors*. Consider examples (4) and (5):

(4) He defends his claims well (Steen et al., 2010, p. 13).

(5) Sometimes it’s like someone took a knife, baby, edgy and dull, /And cut a six-inch valley through the middle of my soul (Steen et al., 2010, pp. 10–11).

Whereas in example (4) the metaphor-related word^8^ *defends* realizes an indirect contrast and comparison between the word’s basic and contextual meaning (physical defense vs. rhetorical defense), the metaphor-related words in example (5) (*took*, *knife*, *edgy*, *dull*, *cut*, *six-inch*, *valley*, *through*, *middle*) do not stage this incongruity (their basic and contextual meanings are identical). Conversely, their metaphorical force derives from a semantic transfer between the conceptual domains of physical and emotional pain, which is expressed “directly.”

To accommodate their procedure to such direct realizations of cross-domain mappings, MIPVU devised a separate set of guidelines (see Steen et al., 2010, pp. 14–15 for the complete exposition). For now, it is important to highlight that word-clusters realizing a cross-domain mapping in a direct manner are detached into their constituent lexical units and treated as an array of discrete direct metaphors within the procedure, to be admitted separately in the database (e.g., *summer’s / day*).

### 2.2. MIP(VU)‘s standardized level of analysis: the lexical unit

The rationale behind choosing the lexical unit as standardized level of analysis is twofold.

A first rationale is theoretical, and relates to the assumption that there exists a functional relationship between words, concepts and referents, in the sense that “most words may be assumed to activate concepts in memory which postulate referents in discourse” (Steen et al., 2010, p. 12). The second motivation is more pragmatic in nature, and involves the creators’ desire to adopt a “maximal approach” to metaphor identification (Pragglejaz Group, 2007, p. 2), and to capture as many layers of metaphorical meaning as possible. Hence their decision to split word-clusters into their component parts, so that all of them may be considered for potential metaphoricity (Pragglejaz Group, 2007, p. 2).

One can readily appreciate the promised advantages that come with a standardized level of analysis. For one, a stable unit of analysis is said to facilitate across-language and study comparison (e.g., Steen, 2017, p. 80). Furthermore, it is taken to guarantee the reliability of subsequent quantitative analyses of the data (Steen et al., 2010, p. 27; Steen, 2017, p. 83).

However, a major issue concerns the aspect of non-compositionality. That is, the recognition that on many occasions the meaning of word-clusters does not derive from the sum of the meanings of its constituent elements but is evoked by the schema which brings them together (see also Fillmore et al., 1988; Goldberg, 1995, 2006; Croft, 2001; Sullivan, 2007). The idea of non-compositionality is recognized to some extent within the protocol (*cf.* Section 2.1), as it is meant to define the demarcation of the lexical unit. Yet, paradoxically, in practice many multiword configurations traditionally considered non-compositional (e.g., Boas, 2005; Stefanowitsch, 2006) – such as classical idioms,^9^ collocations, sayings and other kinds of *listemes* (Di Sciullo and Williams, 1987) – are broken down and dealt with as discrete lexical units within the procedure. For instance, idioms of the type *to wear the mantle* (*cf.*, supra, Section 2.1) are spliced into word-atoms, and treated as individual lexical units (e.g., *to / wear / the / mantle*). This begs the question: how does the MIP(VU) identification-instrument factor in the idea of non-compositionality when dividing multiword segments into lexical units? And what are the reasons for splitting up seemingly non-compositional lexical strings?

### 2.3. Dealing with non-compositionality

When confronted with lexical strings generally considered non-compositional, MIPVU roughly deals with them in one of the following two ways.

In cases where the multiword expressions designate a single referent and are considered sufficiently conventionalized as one unit (Pragglejaz Group, 2007), they recognize the non-compositionality of the string and code is as one lexical unit. A multiword unit is taken as sufficiently conventionalized when it (i) receives a separate POS-tag in the corpus of choice, (ii) is admitted as a single entry in their chosen reference dictionary and, as is the case for compound nouns, (iii) when it conforms to a particular stress pattern (*cf.*, Steen et al., 2010, pp. 27–32). Examples of multiword expressions treated as a unique lexical unit include a finite set of polywords such as *by means of* (Steen et al., 2010, p. 27) and *let alone* (Pragglejaz Group, 2007, p. 4), as well as compound nouns found in the dictionary which carry a primary stress on the first word and a secondary stress on the second, like *power plant* (Steen et al., 2010, p. 31).

On other occasions, the non-compositionality of a lexical string is acknowledged but it does not affect grouping-decisions. Examples include idioms (e.g., *to/ spill / the / beans*; Steen, 2017, p. 80), proper names (e.g., *New / York / Herald / Tribune*; Steen et al., 2010, p. 31), frequent collocations (e.g., *staking / a / claim*; Pragglejaz Group, 2007, p. 27) as well as a great many compound nouns^10^ (e.g., *nuclear/power*; Steen et al., 2010, p. 31). Besides the assumption about the functional relationship between words, concepts and referents, reference is made to the existence of psycholinguistic evidence (i.e., Gibbs, 1994) that, even in such non-compositional expressions, people can find metaphoricity at the word-level (Pragglejaz Group, 2007, p. 27).

In summary, and to conclude Section 2, up until now we have seen that MIP(VU) requires metaphor scholars to divide lexical strings that sit on a continuum ranging from non-compositional (e.g., idioms and frequent collocations) to compositional (e.g., analogies, similes and phrases), and to treat their constituent parts as individual lexical units. Therefore, to disambiguate MIPVU’s idiosyncratic operationalization of “lexical unit” as “metaphor-related words” from the more conventional lexicographer’s interpretation, we will henceforth refer to these as “lexemes.” These units, in turn, are then to be judged for the presence of metaphorical meaning.

However, the identification of linguistic metaphors represents for many scholars only the starting point for subsequent analyses. Often times this includes a semantic-field analysis, with the intention to detect which semantic frames are evoked by the identified metaphor-related words (e.g., Sullivan, 2007), and to be able to answer a wide variety of research questions which require postulating underlying conceptual (Deignan, 2016) or systematic metaphors (Maslen, 2017). In addition, scholars often wish to quantify the results of this semantic analysis (e.g., Catalano and Mitchell-McCollough, 2019), so as to obtain a general picture of frequencies and distributions of detected metaphorical domains, and/or for comparison purposes (e.g., between different languages, language varieties, or speech communities, or between distinct discourse genres, registers, sources, etc.). However, when using the identified metaphorically-motivated words as the input for a subsequent quantitative semantic field analysis, the analyst may run into a host of problems, related to the default level of analysis: the lexeme. These problems are further specified in the next section.

## 3. Problematizing the lexeme

To illustrate the difficulties which the lexeme may cause, we will draw on corpus examples derived from a case study exploring the metaphorical representation of the Deferred Action For Childhood Arrivals (DACA) policy issue in the US written press. Before moving on to the crux of this section (3.2), we will therefore first briefly introduce the case study from which our data stems (3.1).

### 3.1. Research context: introduction of the DACA-case study and data

#### 3.1.1. Aim of the study

The case study which informs this paper encompasses a corpus-based analysis of the news discourse of El Diario, a Spanish-language US-based newspaper. In concrete we are interested in its coverage of the migration debate surrounding the DACA-program, a policy issue which has attracted a great deal of media attention in recent years.

Short for “Deferred Action for Childhood Arrivals,” DACA entails an Obama-era migration program conferring temporary social and legal rights to an undocumented segment of the US-population brought to the country as children, including protection from deportation and a work permit (Walters, 2017). In the public sphere its beneficiaries are known as *Dreamers* (Chávez, 2013). While widely enjoying popular and bipartisan support (Krogstad, 2020), the program has been dragged to Court numerous times by conservative-leaning politicians ever since the Trump administration announced its plans to phase out DACA in 2017. Particularly after a conservative judge (judge Hanen) ruled DACA “illegal” (Aug. 2021) and the Fifth Court of Appeals followed suit (Aug. 2022), the prospects for the program’s future and the Dreamers have never looked more grim^11^.

Our case study’s general aim can be encapsulated by the following research question: how does El Diario use metaphors to frame the DACA-debate? More specifically, which metaphors are deployed to refer to or characterize recurrent referential categories – i.e., discourse actors (e.g., Dreamers, Biden, Trump, judge Hanen, etc.), entities (e.g., DACA), actions (e.g., deportation, regularization, restrictive migratory actions and court rulings), attributes (e.g., legal status) and relationships (e.g., Trump vs. Dreamers) – in this debate?

The research design incorporates a qualitative and a quantitative component. From a qualitative point of view, it seeks to chart which semantic fields (e.g., WAR, MOVEMENT) are used to frame the DACA-debate and how these fields are used situationally^12^ (Van Teeffelen, 1994). From a quantitative perspective, it aspires to measure which metaphorical fields are most productive. This research fits within a rich tradition of (critical) discourse scholarship concerned with the metaphorical representation of social questions in the press (e.g., Arrese, 2015; Nerlich, 2015), such as migration (e.g., Charteris-Black, 2006; Musolff, 2015; Piñero Piñero et al., 2015; Mujagić, 2018; Arcimaviciene, 2019; Montagut and Moragas-Fernández, 2020).

#### 3.1.2. The data

In total, our corpus bundles 25 DACA-related articles published in El Diario during the presidency of Joe Biden (November 3, 2020 - present), amounting to 14.343 words in total. From these 25 articles, 9 were published in the El Paso edition of the newspaper (6,002 words) and 16 in the Juárez version (8,341 words).

The metaphors were identified by running a reduced version of MIP(VU) (Pragglejaz Group, 2007; Steen et al., 2010; *cf.*, supra), meaning that only content words were considered for analysis (7,772 words). As prescribed by MIP(VU) (*cf.*, supra), we selected two dictionaries to aid us with the disambiguation of lexemes basic meanings, namely: *Diccionario del Español de México* (El Colegio de México, A.C, n.d.) and *Diccionario De Uso del Español* (Moliner, n.d.)

After running the MIP(VU) procedure, all linguistic metaphors along with their extended contexts were migrated to a separate spreadsheet in Excel. Our final DACA-database envelops a total of 1,353 (potentially)^13^ metaphor-related words (see Table 1 below).

**Table 1:** Breakdown of the number of analyzed and (potentially) metaphor-related words per local edition.

**Edition**	**# Cases analyzed (content words)**	**# (Potentially) metaphor-related words**
El Paso	3,270	570
Juárez	4,502	783
	Total: 7772	Total: 1353

#### 3.1.3. Data analysis

This dataset, in turn, served as the input for the (manual) annotation of relevant linguistic and context-related variables, such as “semantic field” and “referent/topic.”

The semantic-field analysis of the identified items proceeded inductively, and was carried out at two levels of inference (*cf.*, LeCompte and Schensul, 2013). In the first round of analysis, low-inference descriptors were formulated. At this stage, we remained as close as possible to the words used in the news texts (*cf.*, Cameron et al., 2010). Consider example (6):

(6) … exigió que el Congreso encontrara un camino a largo plazo, como un camino hacia la ciudadanía para inmigrantes indocumentados y beneficiarios de DACA (Diario de El Paso [García], 2021).

(*‘.. demanded that Congress find a long-term pathway, such as a pathway to citizenship for undocumented immigrants and DACA recipients’*)

In (6), the linguistic metaphor *camino (‘pathway’)* was annotated as *Pathway* during the first round of inductive, low-inference coding (as opposed to coding it using a high-inference descriptor, say, *Journey*).

In the second round of coding, and in an additional parameter, we attempted to formulate high-inference descriptors, based on generalizations which were warranted by emerging patterns in the data. For instance, the same token *camino* (*pathway*) was coded as *Journey* after observing that a set of semantically-related, low-level codes such as *Movement toward a destination*, *Trajectory*, *Movement toward a source*, *Destination* were used systematically to frame the DACA-debate.^14^

For the annotation of the linguistic metaphors’ referents, a list was composed (inductively) after reading through the news texts which enumerates recurrent discourse actors, entities, actions, attributes and relationships within the DACA-debate^15^ (referred to henceforth as ‘referential categories’). This list was then used to code the linguistic metaphors (e.g., *a pathway to citizenship*) in our database (for a similar approach, see Cameron et al., 2009; Cameron and Maslen, 2010). Importantly, given that a single metaphor can be used to frame multiple relevant referents simultaneously, we multiplied such metaphors in our database to capture all layers of meaning (*cf.*, ‘multidimensional analysis’; De Backer and Enghels, 2022).

### 3.2. The lexeme as starting point for metaphor analyses: setting out the core problem and related methodological challenges

In what follows, we will sketch the contours of the key problem that the lexeme may pose to metaphor analysis (3.2.1), after which two related methodological challenges will be put at display which can arise when intending to use a database containing atomized metaphor-related words as the starting point for a semantic-field analysis with a quantitative component (3.2.2).

#### 3.2.1. The problem: when the relevant metaphorical image is evoked by a lexical string

The following question emerges: how do we deal with contexts in which the relevant metaphorical image for a particular research topic is evoked by a lexical string, consisting of multiple lexemes?

For clarity’s sake, we have distinguished in our dataset three scenarios in which this tension between lexeme and string is foregrounded: (1) the lexemes involved in a string are individual metaphors; (2) metaphorical lexical strings whose constituent lexemes are not metaphorical in isolation; and (3) metaphorical similes and analogies made out of (in)direct metaphors and non-metaphorical lexemes.

The point of this overview is not to be exhaustive, nor is it to offer individual solutions or an alternative taxonomy to categorize metaphorical lexical strings. Rather, it is meant to report in a structured way some of the contexts in which MIP(VU)‘s focus on metaphor-related words is confronted with difficulties. Simultaneously, it aims to show that this problem does not limit itself to a handful of isolated cases, such as classical idioms of the sort *to wear the mantle* (*cf.*, Section 2), but can instead affect a wide range of multiword units, varying greatly in terms of their length, internal composition and degree of cohesion.^16^

##### 3.2.1.1. Scenario 1: the lexemes involved in a string are individual metaphors

The first scenario embraces linguistic contexts in which the pertinent metaphorical image is elicited by a string of lexemes whose constituent items are different metaphors according to MIP(VU).

An example is the phrase *punto intermedio* in (7), which comments on recent attempts of Democrats to reach a political agreement with the Republican party on the thorny issue of migration reform:

(7) En busca de un [punto intermedio], los demócratas incluyeron una versión inmigratoria “Light” (Diario de El Paso [Zamorano], 2021)

*(‘In search of a [middle ground]*,^17^*the Democrats included a “Light” immigration version’).*

Here, *punto* and *intermedio* are marked as metaphor-related words according to MIP(VU), as both of them possess a sense related to physical space when inspected on a word-by-word basis, which is then mapped onto the more abstract domain of politics. *Punto* evokes the concept of a point on a road, while *intermedio* (*middle*) stirs the mental representation of a physical position in between two points or objects. However, the metaphorical image elicited by the whole differs subtly from that of its individual components. Indeed, the entire expression (*punto intermedio*) calls on the mental picture of a particular *class* of points. That is, not just a point (*punto*) situated anywhere but a point located in *the middle* of a road (un punto *intermedio*).

In the above example, the string *punto + intermedio* thus construes the domain of MIGRATION POLITICS as a PHYSICAL JOURNEY, in which political antagonists (Democrats and Republicans) are presented as occupying a position at opposing ends of the path, and reaching a political consensus on migration reform is presented as meeting each other halfway, on *middle ground*.

The point that we wish to bring home with this example is that – though possible to examine the words on an individual basis – such an analysis is not necessarily relevant for our research purpose. For it is the string *punto intermedio* in its entirety that informs us about how metaphor is used to represent one of the referential categories of interest, namely, the political debate surrounding migration reform.

##### 3.2.1.2. Scenario 2: metaphorical lexical strings whose constituent lexemes are not metaphorical in isolation

The second scenario involves situations in which the metaphorical image is evoked by a lexical string whose constituent parts are not metaphorical in isolation according to MIP(VU), as in (8):

(8) Osmán es un “dreamer” que ha esperado con paciencia el estreno de la carretera que lo lleve de su estado de incertidumbre al de la certeza de la legalización migratoria. Pero no ha [(esperado) con los brazos cruzados]: Obtuvo con mucho esfuerzo y sacrificio una licenciatura, y actualmente se desempeña profesionalmente en una importante empresa de comunicaciones para la comunidad hispana (Diario de El Paso [Zamorano], 2021).

*(‘Osmán is a “dreamer” who has waited patiently for the opening of the road that will take him from his state of uncertainty to the certainty of immigration legalization. But he has not [(waited) with his arms crossed]: He obtained with much effort and sacrifice a bachelor’s degree, and is currently working professionally at a major communications company for the Hispanic community’).*

In example (8), the lexemes which make up the string *(esperar) con los brazos cruzados (waiting with his arm crossed)* are not metaphorical according to MIP(VU), as the contextual meaning of the words *con* (*with*), *brazos* (*arms*) and *cruzados* (*crossed*) cannot be understood in comparison with their more basic meaning (i.e., they are identical; *cf.*, step 4).

However, the string *(esperar) con los brazos cruzados (waiting with his arm crossed)* as a whole can, in fact, by interpreted as metaphorical. As such, it can be said to possess a non-literal meaning related to the realm of mental processes, and is called upon to cast the pro-active attitude of Osmán as a physical action.

At this point it is worth pointing out the affinity of this class of metaphorically-motivated expressions to a particular kind of ‘idiom-like collocations’. These are typically grounded in bodily experience and gestalts, rather than being rooted in analogy, and derive their “metaphorical force from their meaning as a whole, which explains why they cannot be decomposed and why they are relatively fixed syntactically and lexically” (Deignan, 1999, p. 33). An example discussed by Deignan includes the collocate *(take) a deep breath*, which aside from referring to a physical action is said to stand in for a more abstract, psychological sort of preparation. This type of ‘non-intellectual’ mapping gives rise to lexical strings whose metaphoricity is powered by the expression as a whole, not by any of the lexemes in isolation or the sum of its parts.^18^ Hence, all such expressions will inevitably fall within this second scenario, and thus need to be examined in their entirety if we wish to capture their metaphorical quality.

##### 3.2.1.3. Scenario 3: metaphorical similes and analogies made out of (in)direct metaphors and non-metaphorical lexemes

The third scenario includes two types of lexical strings which merit a special mention, namely (metaphorical) similes and analogies. These linguistic realizations of cross-domain mappings can be decomposed, in the terminology of MIP(VU), in a number of discrete *direct metaphors* (*cf.*, Section 2.1). Yet, the metaphorical image of interest might (only) be summoned by the entire expression.

In (9) the Dreamer Osmán uses an analogy to express what it is like to be a DACA-holder:

(9) Algunos activistas consideran que es un pequeño avance, es decir lograr permisos de trabajo y protección contra las deportaciones por cinco años, es mejor que nada. Pero para muchos de estos cientos de miles de jóvenes que han esperado pacientemente, la opción intermedia no es un consuelo. “Para mí no lo es. Es una resolución que tiene fecha de expiración. [Como ir pintando rayas en la pared cada día que pasa],” nos dice Osmán (Diario de El Paso [Zamorano], 2021).

*(‘Some activists believe that it is a small breakthrough,* i.e.*, getting work permits and protection from deportation for 5 years, is better than nothing. But for many of these hundreds of thousands of young people who have waited patiently, the in-between option is no consolation. “For me it’s not. It’s a resolution that has an expiration date. [Like painting stripes on the wall with each passing day],” Osmán tells us’*).

The expression *(Como) ir pintando rayas en la pared cada día que pasa* is made up of six direct metaphors (if only counting content words), if we apply the MIPVU method (i.e., *ir*, *pintando*, *rayas*, *pared*, *día*, *pasa*). However, it is the string as a whole which evokes the wall-painting scenario and which is mobilized by Osmán to frame the situation of Dreamers as an emotionally-straining experience. One could even defend the claim that the entire expression calls to mind a PRISON-frame, in which Dreamers like Osmán are cast in the role of prisoners on death parole, condemned to count down on the wall of their cells the days remaining until their execution date.

However, there exists an additional layer of analytical complexity, highly frequent among similes and analogies. That is, some units functioning as *direct metaphor* at a higher level of analysis (*ir, pintando, rayas, pared, día, pasa*) can be interpreted simultaneously as *indirect metaphors* at the level of the lexeme (*cf.*, Section 2.1). A case in point is the lexeme *passing* (*…each passing day*). Aside from participating in the PRISON-scenario, it can be interpreted as a movement-metaphor framing the abstract concept of TIME (instantiated by the lexeme *day*) in concrete terms of MOVEMENT. As evidenced by the cognitive linguistics literature, this comprises a highly-conventionalized manner to think and speak about the progression of time (e.g., Lakoff and Johnson, 1980; Kövecses, 2010). And although this secondary interpretation is also correct and might be of interest for some research endeavors, we might ask ourselves how relevant results obtained at this scale are for the research question at hand (‘How is metaphor used to frame the DACA-debate’?). To what extent does considering analogies and similes at the level of the lexeme comprise a worthwhile enterprise across the board, or does it simply produce noise?

This question concerning the decision of the relevant unit of analysis – which can be said to apply to the whole inventory of lexical strings put at display in Section 3.2.1 – becomes all the more pertinent when we move on to the semantic annotation of the metaphors contained in our dataset and the subsequent quantification of the results, as will be argued in the following section.

#### 3.2.2. Methodological implications: the semantic annotation and quantification of metaphorically-motivated strings built out of multiple lexemes

What are the implications of using lexemes as the default starting point for linguistic analysis, if any? This section shows that the choice of the analytical unit may have several important methodological implications for metaphor scholars intending to use the output of MIP(VU) as the basis for a quantitative semantic analysis. Concretely, these methodological challenges relate to practical decisions that the researcher needs to make regarding the semantic annotation (3.2.2.1) and the quantification (3.2.2.2) of lexemes incorporated in a metaphorical lexical string.

##### 3.2.2.1. Semantic annotation

The first methodological difficulty concerns the semantic annotation of lexemes belonging to metaphorically-motivated lexical strings, which – conform to MIP(VU) – are marked as individual metaphor-related words and thus included separately in the database.

Consider the following sentence from our corpus, which captures a journalist’s evaluation of the situation of the DACA-program (10).

(10) En otras palabras, DACA [pende del hilo] de la incompetencia de la administración Trump (Diario de El Paso [Wilkinson], 2021).

(*‘In other words, DACA [hangs on the thread] of the Trump administration’s incompetence’)*.

In accordance with the guidelines spelt out by MIP(VU), the metaphorically-motivated idiom *pender del hilo de* … can be decomposed into its constituent lexemes, *pender* and *hilo*. Both lexemes, then, are admitted as separate entries into our database, and thus require an individual semantic analysis.

The question arises as how best to carry out a semantic field annotation of such units? The analyst could opt for one of two strategies:

- They may decide to focus on the direct-associated meaning of the lexemes in isolation and annotate them accordingly.

For *pende + hilo*, this could mean that *pende* – whose basic meaning denotes a manner of being positioned in physical space – is assigned the semantic field of LOCATION and *hilo* – a thin thread commonly used for sewing – is categorized within the field of TEXTILE:

- *pende*: LOCATION
- *hilo*: TEXTILE

However, such an atomistic annotation does not prove to be very instructive for the research question guiding our case study. Hence, a case can be made for the idea that, on this occasion, the metaphorical expression *pende del hilo* as a whole comprises the most relevant level of analysis. For it is the phrase in its entirety which underscores the peril of DACA’s situation and evokes the image of a dangerous location in the mind’s eye of the reader: DACA is presented as an object hanging on a thread, which can fall to the ground and burst at any moment. This reading is reinforced by surrounding discourse cues, most notably the description of *the thread* as being made of *the Trump administration’s incompetence*.

This insight may lead the analyst to adopt an alternative, more context-sensitive, strategy:

- The analyst may retain the analytical decomposition of metaphorical expressions in their database but choose to annotate their constituent elements consistently, taking into consideration the meaning of the expression as a whole.

For *pende + hilo*, whose collective, non-literal meaning could be paraphrased as TO BE IN A DANGEROUS LOCATION, this could look like:

- *pende*: TO BE IN A DANGEROUS LOCATION
- *hilo*: TO BE IN A DANGEROUS LOCATION

Even though far from transparent in the dataset, it is this (second) more context-sensitive road, we submit, that yields the most useful results for the purposes of our study.

It may be tempting to conclude, then, that the decomposition of lexical strings into their constituent lexical units can be maintained in the database, at least in practice, as long as the researcher adopts the second analytical strategy. However, things get more complicated when we seek to integrate a quantitative component in our analysis, for then we face a new methodological problem: the risk of distorted quantitative results.

##### 3.2.2.2. Quantification

Regarding the quantification of the annotated semantic fields (or ‘source domains’), several scholars have called attention to the problems that derive from installing the lexeme as the unit of measurement for their particular research aims, as mandated by MIP(VU) (e.g., Charteris-Black, 2014, p. 176; Vogiatzis, 2019, p. 132). To illustrate using our case study, consider a hypothetical scenario in which the semantic annotation has been conducted exclusively at the level of the lexeme. What happens if we want to compute which metaphorical fields are most productive – a central concern for quantitatively-oriented metaphor scholarship (e.g., Charteris-Black, 2014; Arrese, 2015; Catalano and Mitchell-McCollough, 2019)? The issue we then face is: how do we count?

Take once more the example of *pende + hilo* (i.e., *pende* = LOCATION; *hilo* = TEXTILE), marked in the MIP(VU) protocol as two discrete linguistic metaphors. During the analysis stage, do we count these items as individual metaphors realizing different semantic fields? And are these numbers significant to the research question of interest? In what way is it telling that El Diario uses one LOCATION- and one TEXTILE-related metaphor to frame the DACA-program? This does not seem appropriate.

Alternatively, if we have opted for the second, more context-sensitive annotation strategy (i.e., *pende* = TO BE IN A DANGEROUS LOCATION; *hilo* = TO BE IN A DANGEROUS LOCATION), how do we proceed in this scenario? Do we count them double, as two unique linguistic realizations of the same domain? Once more, this seems hardly ideal.

Another example illustrating this difficulty is the collocation *asestar un golpe (‘to strike a blow’*) which in (11) comments on conservative judge Hanen’s restrictive track record. Note that, following the MIP(VU) procedure, *asestar* and *golpe* are analyzed as two discrete metaphor-related words, and thus constitute separate entries in the database:

(11) A pesar de que Hanen ya [asestó un golpe (contra)] las medidas de protección de inmigrantes al fallar en contra de un programa parecido que cobijaba a los padres de los “dreamers” (Diario de Juárez [Agencias], 2021).

(‘*Although Hanen has already* [*struck a blow (against)*] *immigrant protection measures when he ruled against a similar program that covered the parents of “dreamers’*).

The string *asestó + golpe (strike a blow)* as a whole evokes the image of a VIOLENT ACTION, and in this case it is used to reference judge Hanen’s restrictive court decision concerning immigrant protection measures (*medidas de protección de inmigrantes*). However, conform to MIP(VU), the expression is made up of two violence-related lexemes: *asestar* and *golpe*. Does this mean we count them as separate metaphors, although the expression as a whole stands in for a unique referential category (which may be paraphrased schematically as: “a restrictive migratory action”)?

One may argue that the quantification dilemma sketched above should not pose a problem as long as the analyst is transparent about their quantification protocol and remains consistent. However, this stance becomes harder to maintain if the study involves a comparative or variationist aim (e.g., between different languages, or between texts produced by distinct sources or speech communities; e.g., Perrez et al., 2019) – and, hence, finding the most adequate manner to count becomes increasingly important.

When we extrapolate the implications of this discussion beyond our case study, an important issue emerges for metaphor scholars who wish to run a quantitative analysis. If the analyst observes that the studied source draws significantly on a particular domain (e.g., Journey) to frame a unique referential category (e.g., DACA), how can he/she be sure that the observed frequencies reflect real tendencies? It may as well be so that seeming evidence for the existence of a particularly salient domain in reality results from a high share of multiword expressions, such as phrases (e.g.*, curvas cerradas [‘sharp turns’], trincheras de combate [‘combat trenches’], pende del hilo [‘hangs on a thread’], creciente oleada [‘growing wave’]*, *preparar el terreno [‘prepare the terrain’]*, *mirar de reojo [‘glance sideways’], continua operación [‘continuous operation’]*) and sentences (… *DACA ha pasado por una montaña rusa*… *[‘DACA has gone through a rollercoaster’]*), which have been categorized multiple times within the same semantic domain. This quantification dilemma connects to broader challenges within empirical, quantitative approaches to the study of (cognitive) semantics (*cf.*, Glynn, 2010; Jansegers et al., 2015, p. 383), in the context of which it has been pointed out that observed quantitative patterns can sometimes hide or even distort underlying realities.

To conclude, in this Section the case has been made that using a dataset containing atomized lexemes as the input for subsequent quantitative semantic analyses can give rise to methodological difficulties. Granted the wide diversity in research aims and heterogeneity of datasets existing among metaphor research, we therefore posit that it may be more useful for metaphor researchers to take metaphorical strings – on top of, or instead of, metaphor-related words – as their unit of analysis and measurement. From this observation the following question emerges: given the methodological concerns raised in the introduction (*cf.*, Section 1), is it possible to capture relevant metaphorical strings, composed out of multiple lexemes, in a systematic and transparent way? This is the issue which will be covered in the next section.

## 4. Solutions

As the methodological debate reconstructed in the Introduction established: deciding on where to place unit boundaries is a tricky issue (*cf.*, Section 1). In this section we therefore wish to exhibit three methods metaphor scholars may adopt if their research project mandates the analysis and quantification of above-the-word-level units (4.1). Which methodology to choose will ultimately depend on the judgment of the analyst. Methods 1 and 2 are procedural, and consist in fixing *a priori* guidelines to establish where the unit boundaries will fall. As will be shown below for Methods 1 (4.1.1) and Method 2 (4.1.2) respectively, the formulation of these grouping-directives can start from either a semasiological (‘form-first’; = Method 1) or an onomasiological (‘concept-first’; = Method 2) approach (*cf.*, Glynn, 2010, p. 19). Method 3 is bottom-up, and encompasses the post-hoc aggregation of units, in conjunction with the integration of a descriptive parameter meant to document the analyst’s grouping-decisions (4.1.3). Finally, in Section 4.2, two possible orders to incorporate these grouping-procedures within the metaphor identification and analysis cycle will be presented along with their advantages and drawbacks.

### 4.1. Lexical string formation: three methods

#### 4.1.1. Method 1: procedural, semasiological approach

The first method entails formulating at the onset of the project a set of explicit guidelines detailing which combinations of units will be grouped together.

The criteria underlying the specification of which above-the-word level units will be formed can be of various types. For some research designs, it may be sensible to base the grouping-directives on *semasiological criteria*.

For example, the researcher could be interested in capturing a discrete set of formally definable metaphorical strings, on top of, or instead of, lexemes. Take our case study on El Diario’s coverage of the DACA-debate. Based on our knowledge of our research questions and the nature of our data, we might be interested in lexemes for most of the time, but choose to deviate from this default option in a number of formal contexts. The key would then be to establish objective criteria for each of these configurations to decide which lexical strings instantiate them, and can thus be analyzed as a single unit, rather than as a set of atomized lexemes. Take the notion of *collocation*, which can be generally defined as words that tend to co-occur more often than would be expected by chance (e.g., Firth, 1957; Gries, 2013).

- Collocations (e.g., *punto intermedio, asestar un golpe*, *pende del hilo*, *trincheras de combate, (esperar) con los brazos cruzados*)

The notion of ‘collocation’ is notoriously nebulous (*cf.*, Gries, 2013, p. 138) and difficult to operationalize. One way to establish in a more objective manner (i) which word-clusters count as collocations and (ii) where the collocation-boundaries fall is by running a statistically-founded collocational/collostructional analysis (*cf.*, Stefanowitsch and Gries, 2003) using corpus software like SketchEngine and AntConc.^19^ By looking up in a reference corpus of the target language (i.e., Spanish) whether there exists a strong collocational strength (i.e., a significant degree of association; Stefanowitsch and Gries, 2003, p. 217) between certain lexemes, and by implementing a minimum threshold to establish which values can be considered “strong,” this approach could serve as a reproducible benchmark to operationalize this concept. Applied to the expressions *punto + intermedio* and *asestar + golpe* from our case study, for example, this procedure would render the following results. Provided that we set the minimum collocation-threshold at a value of 3 (*cf.*, Ferraresi and Gries, 2011; Treffers-Daller, 2022), the collocational strength between *asestar* and *golpe* would be considered strong (15, 10) and between *punto* and *intermedio* as extremely strong (51, 28).^20^ Hence, these results would warrant the decision to analyze both multiword expressions as strings.

However, while this first method could be well-equipped for some research questions, it is not adequate for the current case study. This is due to the fact that our research is concerned with the metaphorical framing of specific ‘referential categories’ (e.g., Trump, DACA, restrictive migration measures, etc.), and not all lexical strings instantiating one of these categories necessarily manifests a high collocational strength (or vice versa – not all strings qualifying as collocations instantiate categories relevant for our research question). For this reason, the next approach (Method 2) is our preferred strategy.

#### 4.1.2. Method 2: procedural, onomasiological approach

When relevant concepts and categories are clearly defined in advance, it may be more fruitful to fall back on *onomasiological criteria* to formulate grouping directives. This seems to be particularly so for scholars planning to use the output of the identification procedure as the starting point of a metaphor-led discourse analysis (*cf.*, Cameron et al., 2009; Maslen, 2017). For instance, some scholars could be interested in discerning which linguistic metaphors are used to portray a select array of ‘key discourse topics’ (Kittay, 1987; Cameron et al., 2010). To cite an example, some key discourse topics in Cameron (2010) study on responses to the risk of terrorism in the context of the UK included: terrorism (including risks, causes, perpetrators), communication about terrorism (by authorities and the media), and responses to terrorism (by the authorities and other social groups) (Cameron, 2010, pp. 595–596). Research driven by such an objective could use a list of *a-priori* selected ‘key discourse topics’ as a guide to decide on unit-boundaries, fulfilling in this way the function of tertium comparationis. That is, they can serve as objective external categories that allow for comparison across studies and languages. For instance, certain categories might be expressed in one language or corpus by a single lexeme, while in others a lexical string might be employed. By establishing relevant categories in advance for particular research ends (for instance: DACA, Dreamers, restrictive migration measures), the metaphor researcher gains clear guidelines for when to deviate from the MIP(VU) protocol’s directive to focus solely on lexemes (i.e., ‘metaphor-related words’). As mentioned above, this approach is therefore also best suited for the current research design, where certain *a-priori* defined referential categories are the central focus of the analysis. It would run as follows.

Recall that in our case study we wish to uncover how specific referential categories – i.e., recurrent discourse actors, entities, processes, actions, etc. in the DACA debate (*cf.*, 3.1.3) – are framed through metaphors. Before executing the unit grouping operation, we could therefore start by reading through our corpus of news articles to identify common referents, and compile them in a list as in Table 2.

**Table 2:** List with common referential categories and discursive actors, entities, processes, actions and states in El Diario’s news coverage of the DACA-debate.

**Referential categories**	**Discursive actors, entities, processes, actions and states**	**Description**
Immigrants and immigration	Dreamers	Mention of or reference to Dreamers
	Other migrant groups	Mention of or reference to other groups of migrants, including the parents of Dreamers or other undocumented youngsters
	(Im)migration	Mention of or reference to (the topic of) immigration
	Process of (im)migration	Mention of or reference to the process of migrating
Social, political and legal actors	President Trump/Obama/Biden	Mention of or reference to President Trump, Obama or Biden, and their respective administrations
	The Republican or Democrat Party	Mention of or reference to the Republican or Democrat party and the politicians pertaining to them
	Legislative Branch	Mention of or reference to the legislative branch. Includes legislative bodies (Congress, Senate) and their members (Senators, etc.).
	Judicial Branch	Mention of or reference to legal power. Includes legal bodies (such as the Court) and their members (judges, etc.).
Favorable migration measures	DACA	Mention of or reference to the DACA-program
	Favorable migration laws	Mention of or reference to favorable migration laws, real or hypothetical.
	Favorable migration actions	Mention of or reference to favorable migration actions, real or hypothetical. This includes political decisions and judicial actions that are in the advantage of the migrant population (e.g., an advantageous court ruling)
Unfavorable migration measures	Deportation	Mention of or reference to deportation
	Unfavorable migration laws	Mention or reference to unfavorable migration laws, real or hypothetical.
	Unfavorable migration actions	Mention of or reference to unfavorable migration actions, real or hypothetical. This includes political decisions and judicial actions that are in the disadvantage of the migrant population (e.g., a restrictive court ruling).

This collection of referential categories will then be employed to steer the demarcation of analytical units in the stretches of news discourse under scrutiny. The prime directive for deciding how to carve up linguistic utterances is that the unit boundaries should correspond to one of these predefined referential categories. Consider example (12) from our corpus, in which a commentary is made about a restrictive court ruling (*el fallo*) affecting the DACA-program:

(12) El fallo es “una sirena de alarma” para los Demócratas (Diario de Juárez [Associated Press], 2021a)

*(‘The ruling is “an alarm siren” for the Democrats’).*

In this case, it is the metaphorical string *una sirena de alarma* (‘an alarm bell’, or, literally translated, ‘an alarm siren’) in its entirety that is likened to judge Hanen’s negative court ruling (*el fallo = una sirena de alarma*). An analysis of this string at the level of the lexeme (i.e., *sirena / de / alarma*) would therefore yield no interesting results. Given that one of the pre-defined referential categories in our list includes that of *unfavorable migration actions* – which houses judicial actions such as court rulings that are not in the favor of Dreamers – we consider the phrase *una sirena de alarma* as one unit.

One may appreciate an additional benefit coming with this onomasiologically-oriented grouping strategy: it allows the analyst to capture various layers of meaning at work on different levels of linguistic organization simultaneously. Consider example (13), first discussed in section 3.2.2.1:

(13) En otras palabras, DACA pende del hilo de la incompetencia de la administración Trump (Diario de El Paso [Wilkinson], 2021).

*(‘In other words, DACA hangs on the thread of the Trump administration’s incompetence’).*

Here, the metaphor functions on two scales to frame referents found in our list: the DACA-program (instantiated by *DACA*) and President Trump (realized by *the Trump administration’s incompetence*). On the one hand, the Verb Phrase *pende del hilo* frames DACA as an object that is at risk of falling (*DACA hangs on the thread…*). On the other hand, the NP *thread* (*hilo*) – of which it is said that DACA hangs – is presented as a fine thread of poor quality which symbolizes the incompetence of the Trump administration (…*the thread of the Trump administration’s incompetence)*. We can thus divide the stretch of text into the following units:

- VP: *hanging on the thread* (used to frame DACA)
- NP: *the thread* (= the Trump administration’s incompetence)

#### 4.1.3. Method 3: bottom-up approach

It may not be equally feasible or desirable for all investigations to predict which types of linguistic configurations will best be inspected at a level above that of the lexeme, and to formulate straightforward grouping-directives. Moreover, some projects might require more flexibility in their grouping-procedure, allowing the analyst to move between different levels of analysis as they see fit. This seems to be particularly so, for example, when confronted with multiword units of which the operationalization is more complicated (e.g., analogies), or in the case of bottom-up research projects where relevant categories are not defined at the outset but, rather, emerge throughout the coding procedure. For these reasons, the researcher may think it to be more appropriate to adopt an alternative, analytically more open-ended, method.

This third method involves the post-hoc aggregation of units, coupled with the incorporation of a descriptive parameter meant to keep stock of the DNA of these freshly-formed strings. An application of this workaround could run like follows.

We take the lexeme as the default level of analysis, just as MIP(VU) prescribes. At the moment of diverging from this standard, we document the (formal) composition of our chosen unit of analysis.

Consider the following excerpt from the DACA-corpus:

(14) El Gobierno de Joe Biden propuso este lunes una norma que trasladaría (…) a unos 700 mil inmigrantes que llegaron ilegalmente a Estados Unidos cuando eran niños (…) al final de la fila para ser deportados (Diario de Juárez [Associated Press], 2021b).

*(‘The Government of Joe Biden proposed this Monday a rule that would move (…) some 700 thousand immigrants who arrived illegally in the United States when they were children (..) to the back of the line to be deported’).*

In the above example, we want to analyze the string *(trasladaría) al final de la fila* in its totality, as it instantiates a relevant referential category within our research project (a *positive migration action*) and conforms to a recurring pattern in our data (*DACA-holders are systematically presented as being moved by a third party to a physical location*), and thus decide to group its constituent elements together. When pursuing approach 3, this decision would then need to be followed-up by documenting the composition of the newly-formed string, like so:

- *(trasladar) al final de la fila*: VP > (dis)placement verb + loc. Prep. phrase

The addition of such a descriptive variable does not only safeguard the transparency of this strategy, but will also generate interesting results in their own right. As such, it may provide insight into the levels of linguistic organization at which the majority of metaphors finds itself (e.g., the level of the lexical unit, the phrase-level, the sentence-level, the discourse-level), and allow for the calculation of separate frequencies for each of these levels if the researcher requires a stable unit of measurement (*cf.*, Sections 1 and 2). Likewise, it can grant us some sense of the formal constitution of the metaphorically-motivated expressions stored within a dataset. A drawback, however, is that the inclusion of an additional descriptive parameter makes metaphor identification (even) more time-consuming, and might render extra data superfluous for the research objectives of interest.

### 4.2. Integrating the methods within the metaphor identification and analysis cycle: two possible orders

What does the incorporation of one of these methods (procedural-semasiological vs. procedural-onomasiological vs. bottom-up) look like in practice? As mentioned in the introduction, MIP(VU) was initially designed to be methodologically distinct from subsequent semantic coding and quantification procedures. Recognizing that there exists no one-size-fits-all answer suitable for the whole gamut of metaphor scholarship, two possible approaches to integrating the formation of above-the-word level units within the metaphor identification and analysis cycle will be exhibited. To orient the reader in selecting the strategy which will prove most appropriate for their analytical ends, we will signal the advantages and disadvantages attached to each option.

**Figure 1:**
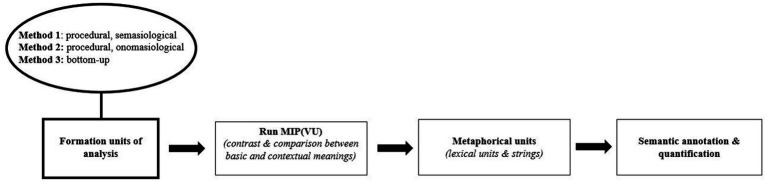
Order 1. lexical string formation after running MIP(VU).

The first possibility is to conduct the above-the-word level aggregation of lexemes *after* performing the MIP(VU) procedure. Concretely, this means grouping the output of MIP(VU) (which includes metaphorical and non-metaphorical lexemes) into metaphorical strings using one of the three methods presented earlier (see Figure 1). In this approach, the creation of metaphorical strings can be integrated into MIP(VU) as an optional extension, serving as a bridge between metaphor identification and the quantitative semantic analysis of detected linguistic metaphors (i.e., metaphorical lexemes and strings).

Aside from being easily compatible with MIP(VU), an advantage of this strategy is that it uses high-quality metaphor-related words and unmetaphorical lexemes, obtained in a statistically reliable fashion (*cf.*, Steen et al., 2010), as the input for subsequent string-formation. One important disadvantage, however, is that this option does not allow the researcher to capture metaphorical strings of which the component parts are not metaphorical in isolation (*cf.*, Section 3.2.1.2; scenario 2 > e.g., *(esperar) con los brazos cruzados*). The time–cost is another obvious drawback.

**Figure 2:**
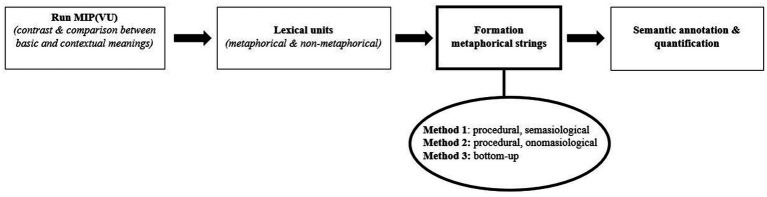
Order 2. unit formation before running MIP(VU).

The second option involves determining relevant units of analysis (i.e., lexemes *and* strings) *before* running the MIP(VU)-protocol, as visually rendered in Figure 2. Within this strategy, the analyst would adopt one of the three grouping-procedures (procedural-onomasiological vs. procedural-semasiological vs. bottom-up) to establish relevant units of analysis, and use the resulting lexical *and* multiword items as the basis for running MIP(VU). Like the core principle underpinning the MIP(VU) procedure dictates, this implies checking whether a more basic meaning can be established which can be understood in contrast and comparison with the unit’s contextual meaning (*cf.*, Section 2).

The edge that this strategy has over the first one is that it enables analysts to capture metaphorical lexical strings whose component parts are not metaphor-related when inspected on an individual basis (*cf.*, Section 3.2.1.2; scenario 2 > *(esperar) con los brazos cruzados*). Moreover, this option is probably more time-efficient as well, given that it collapses step 2 of the MIP(VU)-protocol (‘divide the text under analysis into its constituent lexemes’; *cf.*, Section 2.1) and the above-the-word-level grouping procedure into one single operation.

However, being that the metaphor identification would no longer part exclusively from lexemes, a downside is that dictionaries cannot always serve as a benchmark to establish analytical units’ basic (and potentially contextual) meanings. In case of multiword strings, particularly those which have not been conventionalized (e.g., similes, analogies), researchers would need to rely on a paraphrase test to determine whether the unit under examination can be considered metaphorically-used or not: i.e., is it possible to formulate a more basic meaning of this multiword unit which manifests a similarity and incongruity with the string’s contextual meaning?^21^ Once more, to enhance transparency of the procedure, researcher can choose to record their coding decisions.

## 5. Conclusion

In this paper, we have set out several challenges that the metaphor identification instrument MIP(VU) poses to scholars who are interested in stretches of (metaphorical) language reaching beyond the boundaries of the procedure’s standardized unit of analysis, i.e., the lexeme (Section 2). The main methodological contribution consists in its proposal of a series of flexible guidelines to modify MIP(VU) in such a way as to enable metaphor researchers to (also) capture above-the-word level units in a systematic and transparent fashion (section 4).

In particular, we exhibited three methods which allow for the grouping of above-the-word level units; one is procedural and semasiological (4.1.1), another is procedural and onomasiological (4.1.2) and the final one works bottom-up (4.1.3). Furthermore, two possible orders were presented to incorporate these methods (procedural-semasiological vs. procedural-onomasiological vs. bottom-up) into the metaphor identification and analysis cycle, along with their advantages and drawbacks. As proposed in Section 4.2, metaphor scholars can either carry out (multiword) unit-formation *before* running the MIP(VU) procedure or, alternatively, perform this operation *after* implementing MIP(VU) so as to bridge metaphor identification and analysis.

The adaptable guidelines presented in this paper are both illustrated with data and inspired by insights derived from a case study of a Spanish-language US-based newspaper’s coverage of the migration debate surrounding the Deferred Action for Childhood Arrivals (DACA) program (*cf.*, 3.1). However, we anticipate that these guidelines can be extrapolated to other research contexts where it is important to (also) capture above-the-word-level metaphorical units as well, a need brought to the fore in a multitude of studies (e.g., Sullivan, 2007; Cameron and Maslen, 2010; Charteris-Black, 2014; Vogiatzis, 2019).

As for the limitations of this paper, space constraints prevented us from delving more extensively into the proposed methods and individual strategies (e.g., the collocational strength analysis suggested as part of Method 1, *cf.*, 4.1.1). Future research could explore the different strategies more in-depth, and evaluate empirically how they work out in practice and across languages.

## Data availability statement

The original contributions presented in the study are included in the article/Supplementary material, further inquiries can be directed to the corresponding author.

## Author contributions

LB conceptualized the original idea, analyzed the data, and wrote the manuscript. RE provided important conceptual contributions, gave thorough feedback on different versions, and reduced the wordcount of the final draft of the manuscript. PG pre-processed the data (e.g., the lemmatization and automatic POS-tagging) and gave valuable feedback. All authors contributed to the manuscript and approved the submitted version.

## Footnotes

^1^For an overview, see: Pragglejaz Group (2007, pp. 32–34) and Steen (2007).

^2^Note on terminology: in contexts where it is important to differentiate between the original and the updated protocol, we will, respectively, employ “MIP” and “MIPVU” to signal this distinction. On all other occasions, we will use the acronym “MIP(VU)” to refer to the procedure.

^3^As will be touched upon in Section 2, *(non-)compositionality* denotes the idea that the meaning of an expression as a whole cannot be derived from the sum of the meaning of its parts. Importantly, (non-)compositionality is a matter of degree rather than implying a categorical distinction between *compositional* and *non-compositional* expressions (e.g., Cuenca and Hilferty, 2007).

^4^According to Deignan (1999), the collocation *hit hard* possesses a non-literal meaning related to the domain of PHYSICAL VIOLENCE, which is mapped onto the target domain of EMOTIONS (p. 22).

^5^Pinker (2015) foregrounds that the locative meaning encoded by the entire prepositional phrase *on top of the world* is mapped onto the more abstract domain of EMOTIONS. Interestingly, as Pinker highlights: such “use of prepositional phrases as if it was an adjective to describe a mental or emotional state follows a common pattern in English” (p. 396).

^6^Sullivan (2007) posits that it is the sentence *Two people are chasing the same world title* as a whole which evokes the Object Event-Structure metaphor TRYING TO ACHIEVE A GOAL IS HUNTING (p. 108).

^7^Note that it is not our intention to give an exhaustive display of the protocol.

^8^The technical term ‘metaphor-related word’ is introduced in the MIPVU procedure to encapsulate both ‘direct’ and ‘indirect’ manifestations of metaphor in language. It is used interchangeably with the original MIP procedure’s notion of ‘lexical unit’ (which, as we will see in Section 2.3, is operationalized within MIP(VU) as individual lexemes).

^9^Important to point out is that a growing body of linguistic and psychological literature has nuanced this assumption, drawing attention to the fact that idioms constitute a heterogenous group of expressions whose members can be placed on a *continuum* of compositionality (*cf.*, Cuenca & Hilferty, 2007, pp. 116–121).

^10^Compound nouns which are admitted in the dictionary as a unique entry are to be broken down if they do not match a specific stress pattern, even though they stand in for a unique referent (*cf.*, Steen et al., 2010, p. 31).

^11^For an overview of DACA’s history and the context of its creation, we direct the reader to Chomsky (2014, pp. 152-180) and Chávez (2013, pp. 181–208). For a timeline detailing more recent developments of the program, see: American Immigration Council (2021).

^12^Following Van Teeffelen (1994), the notion *situational use* is adopted to refer to a single metaphor’s variable usage, meaning and function depending on the context in which it is deployed. For example, WAR-metaphors may convey either a negative or, alternatively, a positive, empowering meaning depending on their discursive context (*cf.*, Boeynaems et al., 2017; Semino et al., 2017; De Backer and Enghels, 2022).

^13^In line with Cameron and Maslen (2010), we adopt the term “potentially” to emphasize that no claims are made about the metaphorical value of the identified linguistic expressions for actual speakers/writers and listeners/readers (*cf.*, Cameron and Maslen, 2010, p. 102).

^14^For an overview of the annotation scheme used to code our linguistic metaphors in terms of their semantic fields (low and high inference labels), including examples from our corpus, see the Supplementary materials.

^15^For an overview of the annotation scheme used to code our linguistic metaphors in terms of their referent/topic, including examples from our corpus, see the Supplementary materials.

^16^The point made in this section calls to mind Geeraerts (2002, 2009) prismatic model for the description of semantic specialization in composite expressions (i.e., idioms and compounds). Similar to the argumentation presented here, Geeraerts draws attention to the fact that not all elements of the figurative meaning of composite expressions may be mapped transparently onto the literal meanings of its constituent parts (a principle referred to as “isomorphism”). In contrast to Geeraerts’ taxonomy, which primarily focuses on idioms and compounds, the classification presented here is broader in its scope, extending to a wide range of decompositional multiword expressions (such as analogies, similes and collocations).

^17^Note that the English equivalent of *punto intermedio* (‘middle ground’) – while admitted as a single entry into MIP(VU)‘s reference dictionary (Macmillan) and control dictionary (Longman) – is not counted as one single lexical unit according the MIPVU-guidelines, since it does not conform to the required stress pattern. The Spanish expression *punto intermedio* (literally translated: ‘middle point’) is also not counted as one single lexical unit, given that it is not taken up as a whole in the chosen reference dictionaries.

^18^As a careful reviewer observed, a compositional analysis of the collocate *(take) a deep breath* is also defensible, where *(take) a breath* is mapped onto the concept of ‘regeneration / recovery’ and *deep* specifies that the regeneration / recovery takes time and should be thorough. This observation calls attention to the fact that ‘(de-)compositionality’ is not a binary notion, and that more often than not different analyses of the complex linguistic reality are possible.

^19^For inspiration on corpus-linguistic approaches to metaphor analysis, see Deignan (2005). For more information on the notions of ‘collocations’ and ‘collostruction’, and (quantitative) corpus-driven approaches to collocational/collostructional analysis, see, e.g., Stefanowitsch and Gries (2003).

^20^These measures are based on a 200-token sample of V + N (*asestar + golpe*) and a 200-token sample of N + ADJ (*punto + intermedio*), which were gathered from the Spanish reference corpus of the corpus-software SketchEngine. The collocational strength analysis was carried out in R. The association measure used was the Fisher-Yates exact (one-tailed). See the Supplementary materials for the samples and script used.

^21^At this point it is important to set aside a red herring. Using a paraphrase test to probe whether it is possible to formulate a more basic (and potentially, a contextual) meaning which can be understood in contrast and comparison with one another does *not* mean that the metaphor identification and analysis stage are lumped together (*cf.*, Section 1). For it does not require the analyst to make a final decision about the nature of the underlying mapping. Note that the MIP(VU)-procedure similarly does not require the researcher to make a decision concerning which of the sense description detailed in the reference dictionary can be considered as its basic meaning (and contextual meaning). Words are marked as metaphor-related if the researcher can detect *one or more* basic meanings that *could* be understood in contrast and comparison with the contextual sense of the word. The annotation of semantic domains is reserved for a subsequent stage of analysis, and falls outside the purview of MIP(VU) (*cf.*, Steen et al., 2010; Nacey et al., 2019). The same holds true for the proposed paraphrase test to test for the (potential) presence of metaphorical meaning.

## References

[ref2] American Immigration Council. (2021). Deferred action for childhood arrivals (DACA): an overview. Available at: https://www.americanimmigrationcouncil.org/research/deferred-action-childhood-arrivals-daca-overview

[ref3] Arcimaviciene L. (2019). “Gender, metaphor and migration in media representations: discursive manipulations of the other” in Migration and media: Discourses about identities in crisis. eds. Viola L. Musolff A. (Amsterdam: John Benjamins), 137–159.

[ref4] Arrese A. (2015). Euro crisis metaphors in the Spanish press. Commun. Soc. 28, 19–38. doi: 10.15581/003.28.2.19-38

[ref10] Boas H. C. (2005). Constructional idioms and periphrasis: the progressive construction in English. Cogn. Linguist. 16, 463–490.

[ref11] Boeynaems A. Burgers C. Konijn E. A. Steen G. J. (2017). The impact of conventional and novel metaphors in news on issue viewpoint. Int. J. Commun. 11, 2861–2879.

[ref12] Cameron L. (2010). Responding to the risk of terrorism: the contribution of metaphor. DELTA 26, 587–614. doi: 10.1590/S0102-44502010000300010

[ref13] Cameron L. Low G. Maslen R. (2010). “Finding Systematicity in metaphor use” in Metaphor analysis. Research practice in applied linguistics. eds. Cameron L. Maslen R. (Sheffield: Equinox), 116–146.

[ref14] Cameron L. Maslen R. (2010). “Identifying metaphors in discourse data” in Metaphor analysis: Research practice in applied linguistics, social sciences and the humanities. eds. Cameron L. Maslen R. (Sheffield: Equinox), 97–115.

[ref15] Cameron L. Maslen R. Todd Z. Maule J. Stratton P. Stanley N. (2009). The discourse dynamics approach to metaphor and metaphor-led discourse analysis. Metaphor. Symb. 24, 63–89. doi: 10.1080/10926480902830821

[ref17] Catalano T. Mitchell-McCollough J. (2019). “The representation of unaccompanied migrant children from Central America in the United States: media vs. migrant perspectives” in Migration and media: Discourses about identities in crisis. eds. Viola L. Musolff A. (Amsterdam: John Benjamins), 239–262.

[ref18] Charteris-Black J. (2006). Britain as a container: immigration metaphors in the 2005 election campaign. Discourse Soc. 17, 563–581. doi: 10.1177/0957926506066345

[ref19] Charteris-Black J. (2014). Analysing political speeches: Rhetoric, discourse and metaphor. Basingstoke, Hampshire: Palgrave MacMillan

[ref20] Chávez L. (2013). The Latino threat: Constructing immigrants, citizens, and the nation 2nd. Redwood City, CA: Stanford University Press

[ref21] Chomsky A. (2014). Undocumented: How immigration became illegal. Boston: Beacon Press

[ref22] Croft W. (2001). Radical construction grammar: Syntactic theory in typological perspective. Oxford: Oxford University Press

[ref23] Cuenca M.J. Hilferty J. (2007). Introducción a la lingüística cognitiva. (4th printing) Barcelona: Editorial Ariel, S.A.

[ref24] Dawkins R. (2016). The selfish gene (40th anniversary ed.). Oxford: Oxford University Press.

[ref25] De Backer L. Enghels R. (2022). The persuasive potential of metaphor when framing Mexican migrants and migration. A comparative study of the US written press. Metaphor Social World 12, 204–223. doi: 10.1075/msw.21001.bac

[ref505] Diario de El Paso [García, U.J.]. (2021). Boquea juez texano nuevas solicitudes de ‘dreamers.’ El Diario de El Paso. Available at: https://diario.mx/el-paso/bloquea-juez-texano-nuevas-solicitudes-de-dreamers-20210716-1819264.html

[ref503] Diario de El Paso [Wilkinson, F]. (2021). El sueño americano todavía elude a los Dreamers. El Diario de El Paso. Available at: https://diario.mx/opinion-el-paso/el-sueno-americano-todavia-elude-a-los-dreamers-20201211-1740600.html

[ref504] Diario de El Paso [Zamorano, J.L]. (2021). Una nueva decepción migratoria. El Diario de El Paso. Available at: https://diario.mx/opinion-el-paso/una-nueva-decepcion-migratoria-20211109-1860740.html

[ref506] Diario de Juárez [Agencias]. (2021). Busca grupo de fiscales disolver DACA. El Diario de El Paso. Available at: https://diario.mx/estados-unidos/busca-grupo-de-fiscales-disolver-daca-20210126-1755843.html

[ref501] Diario de Juárez [Associated Press]. (2021a). Piden a Biden y demócratas actuar ante fallo contra el DACA. El Diario de Juárez. Available at: https://diario.mx/estados-unidos/piden-a-biden-y-democratas-actuar-ante-fallo-contra-el-daca-20210717-1819355.html

[ref502] Diario de Juárez [Associated Press]. (2021b). Propone EU norma para proteger a dreamers. El Diario de Juárez. Available at: https://diario.mx/estados-unidos/propone-eu-norma-para-proteger-a-dreamers-20210927-1845748.html

[ref26] Deignan A. (1999). Linguistic metaphors and collocation in nonliterary Corpus data. Metaphor. Symb. 14, 19–36. doi: 10.1207/s15327868ms1401_3

[ref27] Deignan A. (2005). Metaphor and Corpus linguistics. Amsterdam: John Benjamins

[ref28] Deignan A. (2016). “From linguistic to conceptual metaphors” in Routledge handbook of metaphor and language. eds. Semino E. Demjén Z. (London/New York: Routledge), 102–116.

[ref29] Di Sciullo A.M. Williams E. (1987). On the definition of word. Cambridge: MIT Press.

[ref30] El Colegio de México, A.C (n.d.). Diccionario del Español de México (DEM). Available at http://dem.colmex.mx

[ref31] Ferraresi A. Gries S.T. (2011) Type and (?) token frequencies in measures of collocational strength: Lexical gravity vs. a few classics. Paper at Corpus Linguistics 2011, University of Birmingham, 21 July 2011

[ref32] Fillmore C. J. Kay P. O'Connor M. C. (1988). Regularity and idiomaticity in grammatical constructions: the case of let alone. Language 64, 501–538. doi: 10.2307/414531

[ref33] Firth J.R. (1957). Papers in linguistics 1934–1951. Oxford: Oxford University Press

[ref34] García U.J. (2021). Bloquea juez texano nuevas solicitudes de ‘dreamers’. El Diario de El Paso. Available at: https://diario.mx/el-paso/bloquea-juez-texano-nuevas-solicitudes-de-dreamers-20210716-1819264.html

[ref35] Geeraerts D. (2002). “The interaction of metaphor and metonymy in composite expressions” in Metaphor and metonymy in comparison and contrast. eds. Dirven R. Pörings R. (New York: De Gruyter Mouton), 435–468.

[ref36] Geeraerts D. (2009). “Prisms and blends: digging one's grave from two perspectives” in Cognitive approaches to language and linguistic data. Studies in honor of Barbara Lewandowska-Tomaszczyk. eds. Oleksy W. Stalmaszczyk P. (Berlin: Peter Lang), 87–104.

[ref37] Gibbs R. (1994). The poetics of mind: Figurative thought, language, and understanding. Cambridge: Cambridge University Press.

[ref38] Glynn D. (2010). “Corpus-driven cognitive semantics. Introduction to the field” in Quantitative methods in cognitive semantics: Corpus-driven approaches. eds. Glynn D. Fischer K. (Berlin: Mouton de Gruyter), 1–41.

[ref39] Goldberg A. E. (1995). Constructions: A construction grammar approach to argument structure. Chicago, IL: University of Chicago Press.

[ref40] Goldberg A. E. (2006). Constructions at work: The nature of generalization in language. Oxford: Oxford University Press.

[ref41] Gries S. T. (2013). 50-something years of work on collocations. What is or should be next. Int. J. Corpus Linguist. 18, 137–166. doi: 10.1075/ijcl.18.1.09gri

[ref42] Jansegers M. Vanderschueren C. Enghels R. (2015). The polysemy of the Spanish verb sentir: a behavioral profile analysis. Cognit Linguist. 26, 381–421. doi: 10.1515/cog-2014-0055

[ref43] Kittay E.F. (1987). Metaphor: its cognitive force and linguistic structure. Oxford: Oxford University Press.

[ref44] Kövecses Z. (2010). Metaphor: a practical introduction (2nd ed.). Oxford: Oxford University Press.

[ref45] Krennmayr T. (2013). Top-down versus bottom-up approaches to the identification of metaphor in discourse. Metaphorik.de 24, 7–36.

[ref46] Krogstad J. M. (2020). Americans broadly support legal status for immigrants brought to the U.S. illegally as children. Pew Research Center. Available at: https://www.pewresearch.org/fact-tank/2020/06/17/americans-broadly-support-legal-status-for-immigrants-brought-to-the-u-s-illegally-as-children/

[ref47] Lakoff G. Johnson M. (1980). Metaphors we live by. Chicago, IL: The University of Chicago Press.

[ref48] LeCompte M. D. Schensul J. J. (2013). Analysis and interpretation of ethnographic data: A mixed methods approach 2nd. Walnut Creek, CA: AltaMira Press

[ref49] Maslen R. (2017). “Finding systematic metaphors” in The Routledge handbook of metaphor and language. eds. Semino E. Djemén Z. (London / New York: Routledge), 88–101.

[ref50] Moliner M. (n.d.). Diccionario de uso del español. Madrid: Editorial Gredos.

[ref51] Montagut M. Moragas-Fernández C. M. (2020). The European refugee crisis discourse in the Spanish press: mapping humanization and dehumanization frames through metaphors. Int. J. Commun. 14, 69–91.

[ref52] Mujagić M. (2018). Dangerous waters metaphor in news discourse on refugee crisis. Metaphorik.de 28, 99–131.

[ref53] Musolff A. (2015). Dehumanizing metaphors in UK immigrant debates in press and online media. J. Lang. Aggression Conflict 3, 41–56. doi: 10.1075/jlac.3.1.02mus

[ref54] Nacey S. Dorst A.G. Krennmayr T. Reijnierse W.G. (2019). Metaphor identification in multiple languages: MIPVU around the world. Amsterdam: John Benjamins

[ref55] Nerlich B. (2015). Metaphors in science and society: the case of climate science and climate scientists. Lang. Semiotic Stud. 1, 1–18. doi: 10.1515/lass-2015-010201

[ref56] New York Times. (2021). Avala Cámara baja de EU vía a ciudadanía para dreamers. El Diario de Juárez. Available at: https://diario.mx/estados-unidos/avala-camara-baja-de-eu-via-a-ciudadania-para-dreamers-20210318-1774146.html

[ref57] Ortony A.. (1993). Metaphor and thought (2nd ed.). Cambridge: Cambridge University Press

[ref58] Perrez J. Reuchamps M. (2014). Deliberate metaphors in political discourse: the case of citizen discourse. Metaphorik.de 25, 7–41.

[ref59] Perrez J. Reuchamps M. Thibodeau P.H. (2019). Variation in political metaphor. Amsterdam: John Benjamins

[ref60] Pinker S. (2015). The language instinct. London: Penguin Books.

[ref540] Piñero Piñero G. Díaz Peralta M. García Domínguez M. J. (2015). Argumentación y metáfora en el discurso político en torno a la inmigración. Arbor. 191:a224. doi: 10.3989/arbor.2015.772n2010

[ref61] Pragglejaz Group (2007). MIP: a method for identifying metaphorically used words in discourse. Metaphor. Symb. 22, 1–39. doi: 10.1207/s15327868ms2201_1

[ref62] Semino E. Demjén Z. Demmen J. Koller V. Payne S. Hardie A. . (2017). The online use of violence and Journey metaphors by patients with cancer, as compared with health professionals: a mixed methods study. BMJ Support. Palliat. Care 7, 60–66. doi: 10.1136/bmjspcare-2014-000785, PMID: 25743439 PMC5339544

[ref63] Silvestre-López A. J. Navarro i Ferrando I. (2017). Metaphors in the conceptualisation of meditative practices. Metaphor Social World 7, 26–46. doi: 10.1075/msw.7.1.03sil

[ref64] Steen G. J. (2007). Finding metaphor in grammar and usage. Amsterdam: John Benjamins

[ref65] Steen G. J. (2017). “Identifying metaphors in language” in The Routledge handbook of metaphor and language. eds. Semino E. Demjén Z. (London / New York: Routledge), 73–87.

[ref66] Steen G.J. Dorst A.G. Herrmann J.B. Kaal A.A. Krennmayr T. Pasma T. (2010). A method for linguistic metaphor identification: From MIP to MIPVU. Amsterdam: John Benjamins

[ref67] Stefanowitsch A. (2006). “Words and their metaphors: a corpus-based approach” in Metaphor and metonymy in comparison and contrast. eds. Dirven R. Pörings R. (Berlin: Mouton de Gruyter), 83–105.

[ref68] Stefanowitsch A. Gries S. T. (2003). Collostructions: investigating the interaction of words and constructions. Int. J. Corpus Linguistics 8, 209–243. doi: 10.1075/ijcl.8.2.03ste

[ref69] Sullivan K. (2007). Grammar in metaphor: A construction grammar account of metaphoric language. Doctoral dissertation, University of California.

[ref70] Treffers-Daller J. (2022). The simple view of borrowing and code-switching. Int. J. Biling.

[ref71] Van Teeffelen T. (1994). Racism and metaphor: the Palestinian-Israeli conflict in popular literature. Discourse Soc. 5, 381–405. doi: 10.1177/0957926594005003006

[ref72] Vogiatzis A. (2019). Words in crisis. Metaphor valence in persuasive communication. Doctoral dissertation, Aristotle University Of Thessaloniki.

[ref73] Walters J. (2017). What is Daca and who are the dreamers? The Guardian. Available at: https://www.theguardian.com/us-news/2017/sep/04/donald-trump-what-is-daca-dreamers

